# The 3’UTRs of Myelin Basic Protein mRNAs Regulate Transport, Local Translation and Sensitivity to Neuronal Activity in Zebrafish

**DOI:** 10.3389/fnmol.2018.00185

**Published:** 2018-06-12

**Authors:** Julie Torvund-Jensen, Jes Steengaard, Liselotte B. Askebjerg, Kasper Kjaer-Sorensen, Lisbeth S. Laursen

**Affiliations:** Department of Molecular Biology and Genetics, Aarhus University, Aarhus, Denmark

**Keywords:** myelin, oligodendrocyte, MBP, zebrafish, *in vivo* model, local translation, mRNA transport, CNS

## Abstract

Formation of functional myelin sheaths within the central nervous system depends on expression of myelin basic protein (MBP). Following process extension and wrapping around axonal segments, this highly basic protein is required for compaction of the multi-layered membrane sheath produced by oligodendrocytes. MBP is hypothesized to be targeted to the membrane sheath by mRNA transport and local translation, which ensures that its expression is temporally and spatially restricted. The mechanistic details of how this might be regulated are still largely unknown, in particular because a model system that allows this process to be studied *in vivo* is lacking. We here show that the expression of the zebrafish *MBP* orthologs, *mbpa* and *mbpb*, is developmentally regulated, and that expression of specific *mbpa* isoforms is restricted to the peripheral nervous system. By analysis of transgenic zebrafish, which express a fluorescent reporter protein specifically in myelinating oligodendrocytes, we demonstrate that both *mbpa* and *mbpb* include a 3’UTR sequence, by which mRNA transport and translation is regulated *in vivo*. Further functional analysis suggests that: (1) the 3’UTRs delay the onset of protein expression; and that (2) several regulatory elements contribute to targeting of the *mbp* mRNA to the myelin sheath. Finally, we show that a pharmacological compound known to enhance neuronal activity stimulates the translation of Mbp in zebrafish in a 3’UTR-dependent manner. A similar effect was obtained following stimulation with a TrkB receptor agonist, and cell-based assays further confirmed that the receptor ligand, BDNF, in combination with other signals reversed the inhibitory effect of the 3’UTR on translation.

## Introduction

Myelin is a specialized multi-layered membrane structure, which ensheaths neuronal axons. In the central nervous system (CNS), myelin is produced by oligodendrocytes that extend multiple processes and wrap them around axonal segments. Myelin enables the rapid propagation of action potentials and is essential for the metabolic support and survival of neurons (Yin et al., 2006; Nave, 2010). The myelin membrane has a highly specialized molecular composition, consisting of a high content of lipids and a small number of proteins, with myelin basic protein (MBP) being one of the most abundant (Jahn et al., 2009). MBP is essential for normal myelination as demonstrated by the severe dysmyelinating phenotype of the *shiverer* mutant mouse, which lacks functional MBP (Kimura et al., 1985; Roach et al., 1985). For decades, the scientific consensus has been that the principal function of MBP is to bring the inner leaflets of the myelin sheath together, thereby compacting the myelin membrane (Rosenbluth, 1980; Readhead et al., 1987; Harauz et al., 2009; Vassall et al., 2015). However, increasing evidence of other important functions of MBP in myelinogenesis has emerged more recently. These include a role in regulating the composition and organization of the myelin membrane (Fitzner et al., 2006; Aggarwal et al., 2011; Steshenko et al., 2016), reorganization of the actin cytoskeleton during the myelination process (Zuchero et al., 2015), and a suggested role in cell signaling pathways through interactions with SH3 domain-containing proteins (Harauz et al., 2009).

The multiple functions of MBP suggest that a tight temporal and spatial regulation of expression is required for normal myelination. Such regulation has been shown to be at least partly mediated by mRNA transport (Ainger et al., 1993, 1997) and local translation (Colman et al., 1982). *In vitro* studies have started to delineate the molecular mechanisms involved in controlling the different steps of mRNA transport and local translation (Müller et al., 2013). This has resulted in the identification of two sequence elements of the *MBP* mRNA 3’UTR essential for mRNA transport, the RNA trafficking sequence (RTS), and the RNA localization region (RLR) (Ainger et al., 1997). The RTS has been shown to be bound by the heterogeneous nuclear ribonucleoprotein A2 (hnRNP A2) (Hoek et al., 1998) and CBF-A (Raju et al., 2008), and the interaction with RNA-binding proteins is suggested to be necessary for mRNA transport (Munro et al., 1999). Transport of *MBP* mRNA-containing granules to oligodendrocyte processes is dependent on microtubules (Carson et al., 1997) and the kinesin motor protein Kif1b (Lyons et al., 2009), and recent data also suggest a paradoxical requirement for a dynein/dynactin complex (Herbert et al., 2017).

Similar to mRNA transport, *MBP* mRNA translation is also regulated by cis-regulatory elements (Torvund-Jensen et al., 2014). Several trans-acting factors have been shown to be involved in this regulation, including the dynamic interaction with hnRNP K and hnRNP E1 (Laursen et al., 2011; Torvund-Jensen et al., 2014), and the small non-coding RNA 715 (Bauer et al., 2012; Müller et al., 2015). Furthermore, the microtubule-associated protein, tumor overexpressed gene (TOG) (Francone et al., 2007; Maggipinto et al., 2017) both have a suggested role in the regulation of local *MBP* translation.

How the translation of* MBP* mRNA is coordinated by external signals is still an open question. However, a number of recent experiments have suggested that *MBP* translation may be initiated by local axon-glial signaling events. These include glutamate release by electrically active neurons (Wake et al., 2011) and the interaction of an integrin-contactin complex at the glial membrane with the extracellular matrix molecule laminin and the transmembrane protein L1, expressed at the neuronal surface (White et al., 2008; Laursen et al., 2009, 2011). These events activate the non-receptor Src-family kinase Fyn, which phosphorylates several components of the *MBP* mRNA granules (Lu et al., 2005; White et al., 2008, 2012; Müller et al., 2015). This has been suggested to cause dissociation of the granules and initiation of *MBP* translation (White et al., 2008; Laursen et al., 2011).

Most of the details concerning regulation of *MBP* mRNA transport and translation have been obtained by *in vitro* cell culture studies and have not yet been analyzed *in vivo*. As *in vitro* models have the obvious limitation of not observing the studied molecules or cells in their natural environment, thereby excluding the potential effects of e.g., signaling initiated by axon-glial interaction or contact to surrounding tissues, there is a need for the development of reliable *in vivo* models. Recently, the zebrafish has emerged as a powerful model for studying the myelination process (Preston and Macklin, 2015; Czopka, 2016). However, we do not know to what extend the two paralogs, *mbpa* and *mbpb*, undergo the same post-transcriptional regulation as the mammalian *MBP*, and only few details of the regulation of Mbp expression are known. Therefore, we have used transgenic zebrafish lines to assess the involvement of elements and factors in Mbp expression, both at the transcriptional and translational level. Furthermore, we have used zebrafish to study the subcellular localization of the mRNA, and to identify novel factors involved in the regulation of *mbp* mRNA translation.

## Materials and Methods

### Whole Mount *in Situ* Hybridization

Probes for whole mount *in situ* hybridization (WISH) were amplified from zebrafish cDNA by PCR, cloned into the pGEM-T vector (Promega) and verified by sequencing. Antisense and sense (control) probes were synthesized *in vitro* with SP6 or T7 RNA polymerases (Roche), with the addition of the DIG RNA Labeling Mix (Roche). Probes were purified with the Sigma-Spin Sequencing Reaction Clean-Up Kit (Sigma-Aldrich). WISH was performed as described in Thisse and Thisse (2008), with the addition of 5% dextran sulfate (w/v; Sigma-Aldrich) to the hybridization mix for improved signal-to-noise ratio.

### RT-PCR

RNA from zebrafish larvae or zebrafish tissues was isolated with TRI reagent (Sigma-Aldrich) RT-PCR was performed with the OneStep RT-PCR Kit (Qiagen).

For RT-PCR analysis of Oli-Neu cells, total RNA was purified from a fraction of the transfected cells with the RNeasy Mini Kit (Qiagen) and mRNA levels of *β-actin*, *Dendra2* and *G6pd* were assessed by RT-PCR using the OneStep RT-PCR Kit (Qiagen).

### Dendra2 Reporter Constructs and Translation Assay

The coding sequence of *Dendra2* was subcloned from pDendra2-N (Clontech) to the pcDNA3.1(-) vector. The *mbpa* 3’UTR was amplified by PCR from zebrafish cDNA and inserted downstream of the *Dendra2* gene to generate pcDNA3.1-*Dendra2*-*mbpa*3’UTR. The *mbpb* 3’UTR (NM_001271460.1) was purchased from GenScript and inserted downstream of Dendra2 to generate pcDNA3.1-*Dendra2*-*mbpb*3’UTR. pcDNA3.1-*Dendra2*-*mbpa*RTS, pcDNA3.1-*Dendra2*-*mbpb*RTS and pcDNA3.1-*Dendra2*-DelRTS were generated by PCR and/or OE-PCR, followed by insertion between the *BamHI* and *HindIII* cloning sites of pcDNA3.1(-). All plasmids were verified by sequencing.

Translation assays were performed as described in Torvund-Jensen et al. (2014). Briefly, Oli-Neu cells were co-transfected with Dendra2 reporter constructs and pDsRed-Express (Clontech) using Lipofectamine 2000 (Invitrogen). Cells were allowed to differentiate for 48 h before flow cytometric analysis on a Cytomics FC 500 MPL (Beckman Coulter). The translation ratio was calculated as the mean level of green fluorescent signal divided by the mean level of red fluorescence, to correct for variations in transfection efficiency. The translation ratio of Dendra2 without any regulatory sequences was set to 1.0.

To analyze if BDNF and cAMP-dependent signaling had a direct effect on MBP translation, the translation assay was carried out as described above, however, at 5 h and 24 h post transfection the cells were treated with dbcAMP (1 mM) and/or 20 nM BDNF. Forty-eight post transfection the cells were analyzed by flow cytometry. The relative translation index was calculated as described in Laursen et al. (2011).

### Transgenic Zebrafish

All experiments involving zebrafish were carried out according to Danish legislation and the fish were kept under protocol 2017-15-0202-00098 approved by The Animal Ethics Council. Adult stocks of WT strain AB zebrafish and transgenic lines were maintained at 28.0°C, 14–10 h light-dark cycle on recirculating housing systems. Embryos were raised in E3 buffer (5 mm NaCl, 0.17 mm KCl, 0.33 mm CaCl_2_, 0.33 mm MgSO_4_, 10^–5^ (w/w) methylene blue, 2 mM HEPES, pH 7.0) at 28.5°C. Embryos were sedated in tricaine (150 ng/ml; Sigma-Aldrich) in E3 buffer when required.

The *mbpa* promoter (Jung et al., 2010) was purchased (GenScript) and inserted between the *XhoI* and *BamHI* sites of the *Tol2* vector T2AL200R150G (Suster et al., 2009). cDNAs encoding Dendra2 followed by suggested regulatory sequences from *mbpa* or *mbpb* were inserted after the *mbpa* promoter to generate the transposon-donor plasmids.

Transposase mRNA was prepared by linearizing pCS-T2TP by digestion with *Not*I, followed by gel purification (Qiagen Gel Extraction Kit). mRNA was transcribed from the linearized DNA template with the Sp6 mMessage transcription kit (Thermo Fisher) and purified with the RNeasy Mini Kit (Qiagen). Fertilized eggs were co-injected with 25 pg transposon-donor plasmid DNA and 30 pg transposase mRNA at the 1–2 cell stage. Injected embryos were incubated at 28°C and screened for Dendra2 expression 4 days after injections by fluorescence microscopy. Dendra2-positive larvae were raised to sexual maturity crossed to wild-type. Analyses were performed on heterozygotic larvae, unless stated otherwise.

For analyses of the effect of proposed zebrafish RTS motifs on mRNA transport, Tol2 constructs were injected as described above, and Dendra2-positive larvae were fixed with paraformaldehyde at 78 hpf for further analyses.

To test the effect of signaling pathways activated following enhanced neuronal activity on 3’UTR mediated regulation of MBP expression, transgenic zebrafish larvae* Tg(mbpa:Dendra2-mbpa3’UTR)* or *Tg(mbpa:Dendra2)* were transferred to E3 buffer supplemented with PTZ (2 mM), LM22A4 (10 or 20 μM) or dbcAMP (10 mM) at 72 hpf. At 96 hpf, embryos were anesthetized with tricaine (180 ng/mL), embedded in 4% methyl cellulose with tricaine and assessed by fluorescence microscopy. All images were taken from a lateral view of the spinal cord with anterior being left and dorsal top. Images were captured on a Zeiss Axio Observer.Z1 Apotome 2 microscope.

### Statistical Analysis

Comparisons between groups were made with one-way ANOVA followed by Dunnett’s or Tukey’s Multiple Comparison test. All statistical tests were performed using the GraphPad Prism software. A *p*-value of < 0.05 was considered statistically significant. Data are presented as mean ± SD.

## Results

### The Expression of a Specific mbpa Isoform Is Restricted to the PNS

**Figure 1:**
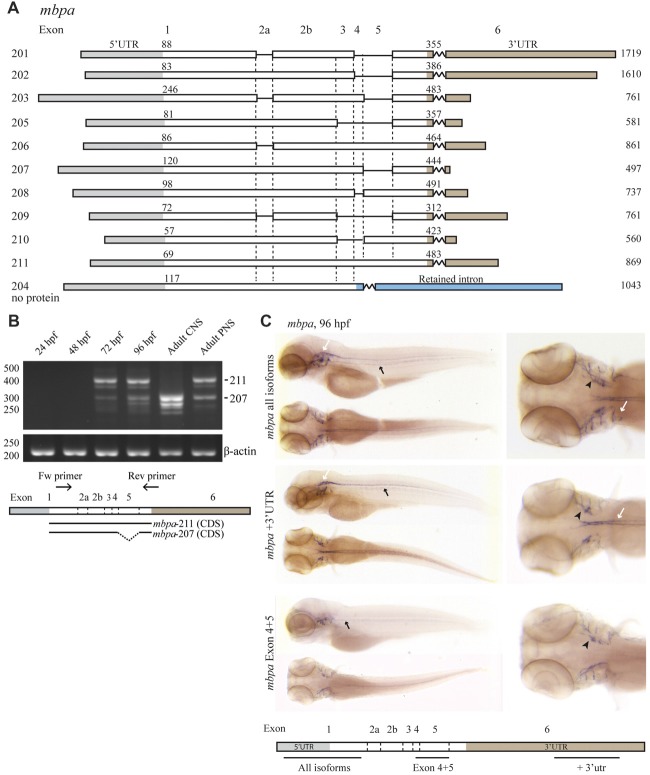
The expression of *mbpa* isoforms is developmental- and tissue-specific. **(A)** Schematic illustration of the *mbpa* isoforms reported in the Ensembl database (release 90). The numbers to the left indicate the identity of the isoform, the numbers above each isoform indicate the position of the first nucleotide in the coding sequence and the 3’UTR, respectively, and the numbers to the right indicate the total reported length. Note that the reported sequences are not complete at the 3’ends. **(B)** RNA was purified from zebrafish larvae at the indicated hours post fertilization (hpf), and from adult brain (central nervous system, CNS) or lateral line peripheral nervous system (PNS). RT-PCR was performed with equal amounts of RNA as template with a primer set annealing to exon-1 and -6. Primers amplifying *β-actin* was included as control. The most prominent DNA bands from the CNS and PNS were sequenced and identified as variants *207* and *211*, respectively. **(C)** Whole-mount *in situ* hybridization (WISH) was performed on zebrafish larvae at 96 hpf with probes annealing to different positions of the *mbpa* mRNA, as indicated. The probes annealing to exon-1 (*mbpa* all isoforms) and exon-6 (*mbpa* +3’UTR) gave similar results, with a distinct signal in the hindbrain and spinal cord of the CNS (white arrows), and in the posterior (black arrows) and anterior (black arrowheads) lateral line system. The probe annealing to exon-4–5 (*mbpa* Exon 4 + 5) resulted in a signal in the posterior (black arrows) and anterior (black arrowheads) lateral line system, however, the signal observed in the CNS with the other two probes was completely absent. Images are representative of several larvae from two independent experiments.

To enable the use of zebrafish as a model for studying MBP mRNA regulation, we first enquired databases and assessed expression experimentally. Two orthologs of mammalian *MBP, mbpa* and *mbpb*, have been identified in zebrafish. For *mbpa*, 11 isoforms are reported in the Ensembl database (release 90, Figure 1A; Yates et al., 2016). The expression of different *mbpa* isoforms was assessed by RT-PCR, with RNA extracted from zebrafish embryos and larvae at different developmental stages as template (Figure 1B). At 24 and 48 hours post fertilization (hpf), no *mbpa* mRNA was detected (Figure 1B). At 72 and 96 hpf, several bands, corresponding to different isoforms, were detected. The onset of *mbpa* expression is in agreement with previously published data (Brösamle and Halpern, 2002; Buckley et al., 2010; Nawaz et al., 2013), and matches the timing of myelination in the zebrafish CNS, which is detectable by electron microscopy at 3 days post fertilization (Buckley et al., 2010). In the adult zebrafish peripheral nervous system (PNS), the expressed *mbpa* isoforms were found to be similar in size to those of the whole larvae at 72 and 96 hpf (Figure 1B). In the adult CNS, however, a different expression pattern was observed: the higher molecular weight bands were completely absent, and the lower bands were more intense, compared with the adult PNS. The most dominant bands in the PNS and CNS were identified by sequencing as *mbpa-211* (exons 1-2a-2b-3-4-5-6) and *mbpa-207* (exons 1-2a-2b-3-4-6), respectively. The expression of *mbpa* isoforms was further investigated by WISH. A probe targeting all isoforms, revealed *mbpa* mRNA expression in the lateral lines and cranial nerves of the PNS, and in the hindbrain and spinal cord of the CNS (Figure 1C). In contrast, when using a probe specific for exon-5, expression was only detected in the PNS (Figure 1C). Combined with the RT-PCR data on the adult tissue this demonstrates a difference in the regulation of *mbpa* mRNA splicing between Schwann cells and oligodendrocytes in zebrafish.

### The Expression of the “Classic” mbpb Isoforms Is Developmentally Regulated

**Figure 2:**
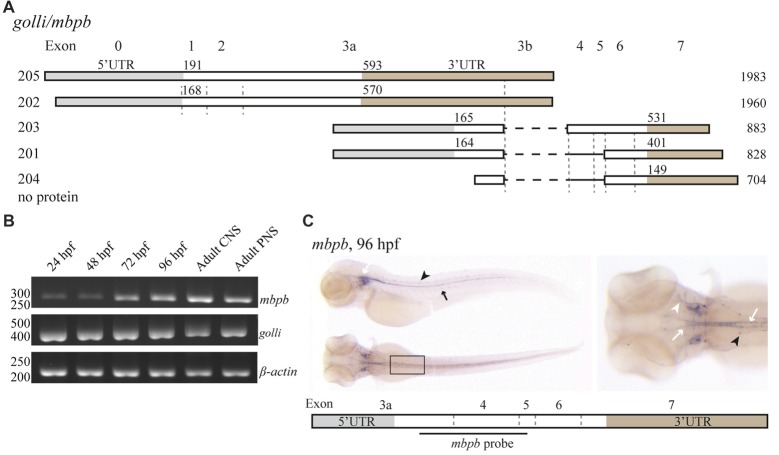
*mbpb* expression is similar, but not identical to *mbpa* expression. **(A)** Schematic representation of the isoforms reported in the Ensembl database (release 90) originating from two different transcription start sites in the *golli/mbpb* gene unit. The numbers to the left indicate the identity of the isoform, the numbers above each isoform indicate the position of the first nucleotide in the coding sequence and the 3’UTR, respectively, and the numbers to the right indicate the total reported length. Isoforms *205* and *202* encode the Golli protein, while the isoforms *203* and *201* encode Mbpb. **(B)** RNA was purified from zebrafish larvae at the indicated hours post fertilization, and from the adult brain (CNS) or lateral line (PNS). RT-PCR was performed with equal amounts of RNA as template with primers recognizing either *mbpb* or *golli* mRNA. A primer set for *β-actin* was included as a control. **(C)** WISH performed on zebrafish larvae at 96 hpf with a probe specific for *mbpb* mRNA resulted in a robust signal in the hindbrain and ventral spinal cord (white arrows), and posterior (black arrow) and anterior (white arrowhead) lateral line system. Furthermore, *mbpb* mRNA was detected in a symmetrical pattern in the dorsolateral spinal cord (black arrowheads). Note the presence of *mbpb* mRNA in the myelin sheaths covering the fine axons travelling laterally from the midline in the hindbrain, best visible in the dorsal view of the head region. Images are representative of two individual experiments with at least 15 larvae examined for each WISH probe in each experiment.

Like *mbpa*, *mbpb* is expressed as several isoforms (Figure 2A). Isoforms *203* and *201* encode protein products similar to Mbpa, while *mbpb* isoforms *205* and *202* encode the Golli protein, which has been assumed to be unrelated to MBP in function (Fulton et al., 2010). The expression of transcription variants *205/202 (golli)* and *203/201 (mbpb)* was investigated by RT-PCR (Figure 2B). The expression of *golli* mRNA was prominent already at 24 hpf, whereas the level of *mbpb* mRNA show a distinct increase from 48 to 72 hpf, similar to what was observed for *mbpa* (Figure 2B). In contrast to *mbpa*, however, there is a weak but detectable signal of *mbpb* mRNA at both 24 and 48 hpf. Both *mbpb* and *golli* mRNAs were detected in the CNS and PNS of the adult fish (Figure 2B). WISH staining for *mbpb* mRNA revealed expression in the hindbrain and spinal cord of the CNS, and in the lateral line and cranial nerves of the PNS (Figure 2C).

### The 3’UTRs of mbpa and mbpb Regulate mRNA Translation in Cultured Cells and *in Vivo*

**Figure 3:**
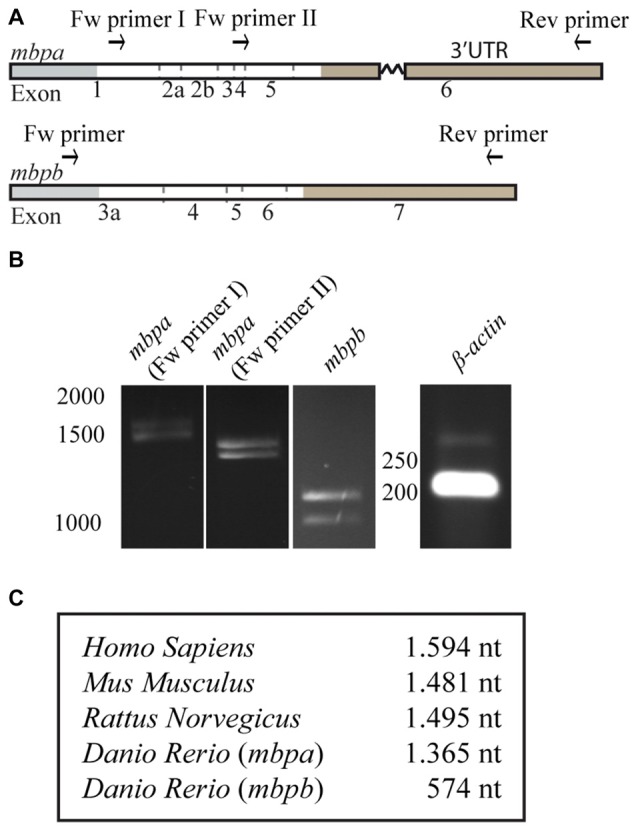
The length of the 3’UTRs is markedly different between *mbpa* and *mbpb*. **(A)** Schematic illustration of the regions recognized by primers used for RT-PCR. **(B)** RT-PCR with RNA extracted from zebrafish larvae at 96 hpf as template. The identity of primers is indicated above the gel pictures. A primer set for *β-actin* was included as a control. **(C)** Table showing the lengths of the 3’UTRs of selected species. For *mbpa* and *mbpb* the lengths of the longest predicted 3’UTR are shown. Results are representative of two individual experiments.

As motifs important for the regulation of MBP expression are found in the 3’UTR of the mRNA in mammals, we asked if the 3’UTRs of *mbpa* and *mbpb* mRNA play a similar role. As the verified sequences of the *mbpa* and *mbpb* mRNAs are not complete at their 3’ends (Figures 1A, 2A), the length of this region was investigated by RT-PCR. By using a forward primer annealing to exon-1 and a reverse primer annealing close to the 3’end of the longest verified 3’UTR of *mbpa*, two PCR products of different lengths were observed (Figures 3A,B). Two products were also detected with a forward primer annealing to exon-4. As the reverse primer binds in exon-6, the products correspond to two isoforms that both include exon-4, but differs in the presence or absence of exon-5, likely to represent the two isoforms identified in Figure 1C (*mbpa-211* and *-207*). Therefore, several of the *mbpa* isoforms contain the full-length 3’UTR. For *mbpb*, RT-PCR data indicate that both isoforms predicted to be protein-encoding (*mbpb-203* and *-201*) include the full-length 3’UTR (Figures 3A,B). When comparing the lengths of the 3’UTRs from *mbpa* and *mbpb* with that from human and rodent *Mbp*, the *mbpb* 3’UTR is remarkably shorter (Figure 3C). This implicates that the transport and translation of *mbpb* could be regulated differently than the other genes.

**Figure 4:**
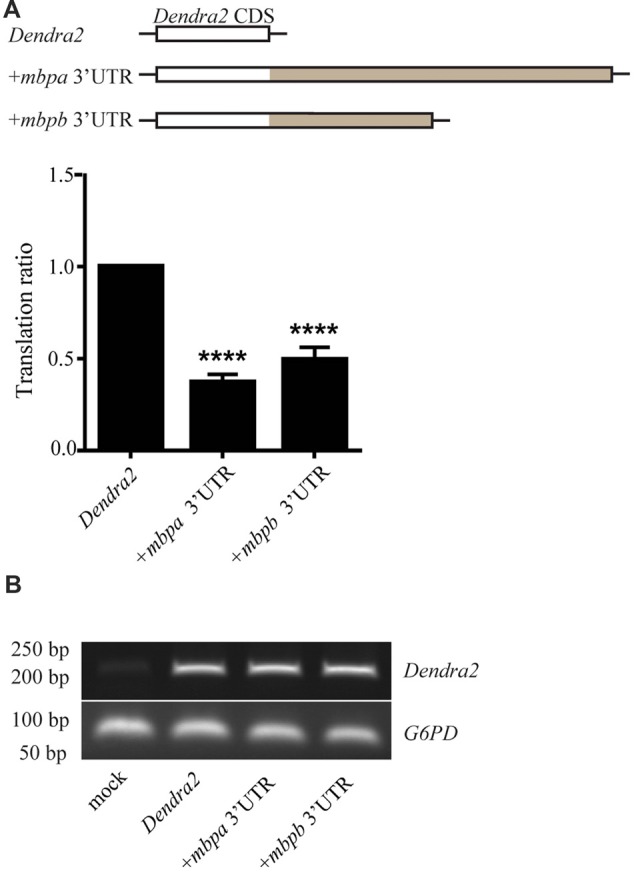
The 3’UTRs from *mbpa* and *mbpb* inhibit mRNA translation in cultured cells. **(A)** The 3’UTR from either *mbpa* or *mbpb* was inserted into a reporter construct containing the coding region of *Dendra2*. The constructs were co-transfected with a DsRed-expressing vector into Oli-Neu cells and protein expression measured by flow cytometry. The relative Dendra2 expression, displayed as the translation ratio, was calculated as described in the “Materials and Methods” section. The results represent the means from three independent experiments ± SD. For each experiment, at least 25 × 10^3^ cells were analyzed. ****p < 0.0001. **(B)** Total mRNA was purified from a fixed number of transfected cells and used as template for RT-PCR with primers recognizing *Dendra2* or the house-keeping gene *G6PD* (control).

**Figure 5:**
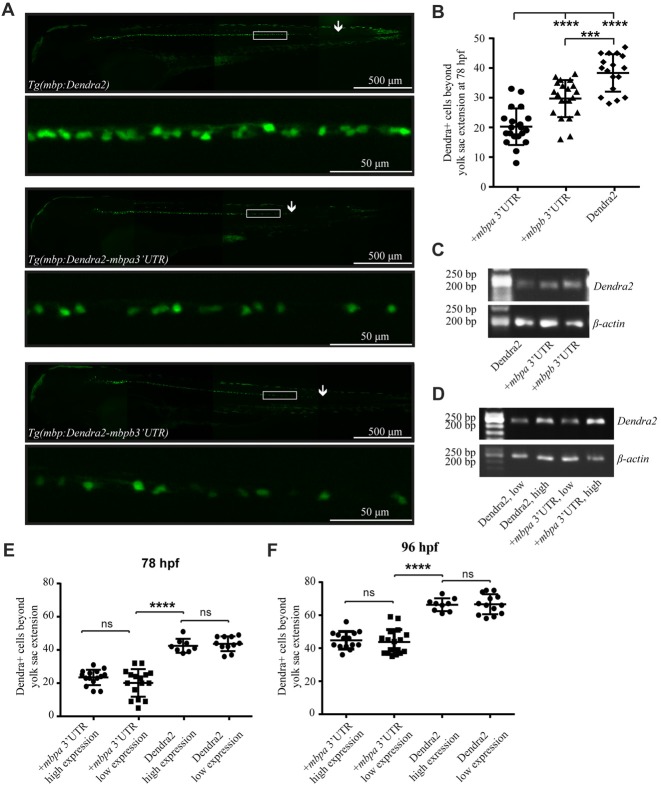
The 3’UTRs from *mbpa* and *mbpb* inhibit protein translation *in vivo*. **(A)** Lateral views of representative full-size transgenic zebrafish at 78 hpf and magnifications of the boxed areas. The most posteriorly located Dendra2-positive cell in each fish is marked with an arrow. **(B)** The number of Dendra2-positive cells caudal to the yolk sac extension was counted at 78 hpf. At least 17 fish were analyzed from each strain. **(C)** RT-PCR with RNA extracted from transgenic zebrafish larvae at 78 hpf as template. Primers recognized *Dendra2* or β-*actin* (control). **(D)** RT-PCR with RNA extracted from transgenic zebrafish larvae grouped into “low expression” and “high expression” based on fluorescent intensity at 78 hpf as template. Primers recognized *Dendra2* or *β*-*actin* (control) **(E,F)** The number of Dendra2-positive cells caudal to the yolk sac extension in transgenic larvae sorted based on the intensity of fluorescence in individual cells at 78 **(D)** or 96 **(E)** hpf. 8–16 fish were analyzed for each group. Results are shown ± SD. ns = not significant, ***p < 0.001, ****p < 0.0001.

To investigate if the 3’UTRs of *mbpa* and *mbpb* regulate protein translation, a construct encoding the sequence of a fluorescent reporter fused to the 3’UTRs from either *mbpa* or *mbpb* was transfected into an oligodendrocyte precursors cell line (Oli-Neu). Including either of the 3’UTRs resulted in a significant decrease in fluorescence signal, compared to cells transfected with a construct without a 3’UTR (Figure 4A). Importantly, the level of mRNA was similar for all constructs (Figure 4B), implicating regulation at the level of mRNA translation. Next, we asked if the *mbpa* and *mbpb* 3’UTRs also inhibited protein translation *in vivo*. To assess this, we analyzed three transgenic zebrafish strains. All strains expressed the fluorescent reporter Dendra2 under the control of the *mbpa* promoter, but differed in the absence or presence of the *mbpa* or *mbpb* 3’UTRs. In zebrafish, the onset of MBP expression and myelination occurs in an anterior to posterior fashion (Almeida et al., 2011). Therefore, we counted the total number of Dendra2-positive cells in the ventral spinal cord caudal to the yolk sac extension and used this to quantify the effect of the 3’UTRs on the onset of protein expression in individual cells (Figures 5A,B). By this approach, we observed a reduced number of Dendra2 positive cells following addition of the *mbpa* or *mbpb* 3’UTR, implicating that the 3’UTRs inhibit the onset of Dendra2 expression. Interestingly, the *mbpa*-3’UTR repressed expression more efficiently than the *mbpb*-3’UTR (Figure 5B). The observed reduction in Dendra2 expression does not correlate with the level of *Dendra2* mRNA, as this was comparable between the strains (Figure 5C), suggesting that the difference in protein expression is a result of translational regulation. Furthermore, the posterior boundary of *Dendra2* mRNA expression was unaffected by the addition of the 3’UTRs (Supplementary Figure S1), further supporting regulation at the post-transcriptional level.

To further verify that variations in mRNA levels did not affect the number of Dendra2-positive cells, heterozygotic crosses were made and the offspring was grouped as “low expression” or “high expression”, based on the intensity of observed fluorescence (Supplementary Figure S2). The expected differences in mRNA levels were verified by RT-PCR, supporting that the two groups represent hetero- and homozygotes (Figure 5D). The number of Dendra2-positive cells caudal to the yolk sac extension in the mRNA “low expression” and “high expression” groups was quantified and the number of cells was found to be similar both at 78 and 96 hpf (Figures 5E,F). This suggests that increased Dendra2 mRNA levels do not affect the onset of protein expression and that oligodendrocytes possess a sufficient excess of factors involved in maintaining translation suppression by the 3’UTR of *mbpa*. In fact, by comparing the number of Dendra2-positive cells in the larvae expressing Dendra2 under the control of the 3’UTR of *mbpa* to those without the 3’UTR at 78 hpf and 96 hpf, we find that that the 3’UTR delays protein expression by approximately 18 h (Figures 5E,F). Importantly, the overexpression of reporter constructs containing the 3’UTRs did not interfere with the ability of oligodendrocytes to myelinate, as the number of internode-like structures generated by individual cells was similar in *Tg(mbpa:Dendra2)* and *Tg(mbpa:Dendra2–3’UTR mbpa)* larvae (Supplementary Figure S3). Taken together, the results obtained both in cultured cells and *in vivo*, strongly supports a role of the 3’UTRs of *mbpa* and *mbpb* mRNA in regulating the onset of protein translation.

### Several Regulatory Elements Are Involved in Targeting mbp mRNA to the Myelin Membrane *in Vivo*

**Figure 6:**
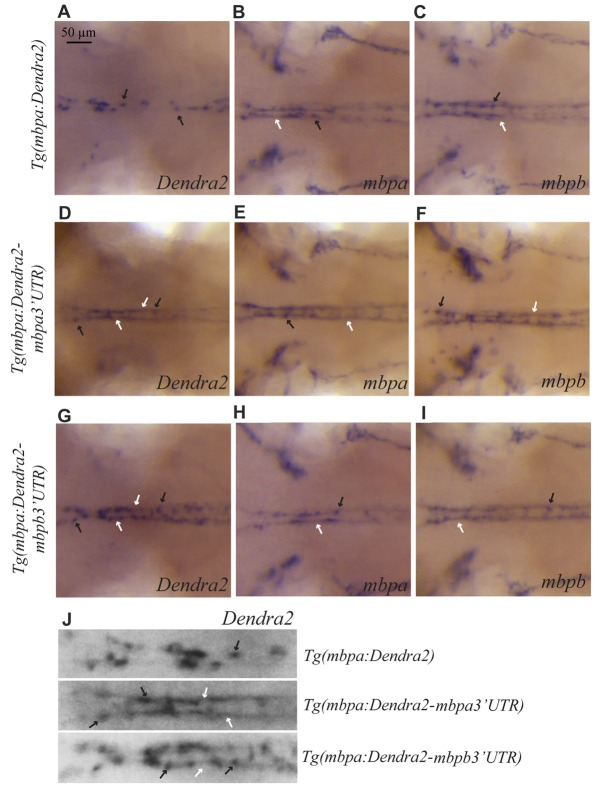
The 3’UTRs from *mbpa* and *mbpb* affect the cellular localization of mRNA. WISH on transgenic larvae with probes specific for *Dendra2* **(A,D,G)**, *mbpa* **(B,E,H)**, or *mbpb* mRNA **(C,F,I)**, as indicated. All pictures represent dorsal views of the neck region. mRNAs localized in cell bodies are indicated by black arrows, and localization in myelin sheaths is marked by white arrows. In the *Tg(mbpa:Dendra2)* larvae, *Dendra2* mRNA is exclusively localized in cell bodies **(A)**. This localization is markedly different from the localization of *Dendra2* mRNA when the 3’UTR from either *mbpa* or *mbpb* is present **(D,G)**. The localization of endogenous *mbpa* **(B,E,H)** and *mbpb* **(C,F,I)** mRNAs is similar in all three transgenic strains. **(J)** Enlarged view of the neck region of Dendra2 WISH of **(A,D,G)**.

Whole mount *in situ* hybridizations on the different transgenic strains was used to test whether the localization of the *mbpa* and *mbpb* mRNAs is regulated by their respective 3’UTRs. In the *Tg(mbpa:Dendra2)* larvae, *Dendra2* mRNA is clearly restricted to oligodendrocyte cell bodies (Figure 6A). This localization is markedly different from that of the endogenous *mbpa* and *mbpb* mRNAs in the same transgenic strain (Figures 6B,C). When the 3’UTR from *mbpa* is added, the localization of the mRNA changes dramatically (Figure 6D). The Dendra2-*mbpa* 3’UTR transcripts can be detected both in cell bodies and in the myelin sheath, in a pattern that is indiscernible from that of the endogenous *mbp* transcripts (Figures 6E,F). The 3’UTR from *mbpb* mRNA also clearly affects the localization of the mRNA (Figure 6G). This demonstrates that despite the pronounced difference in size between the 3’UTRs from *mbpa* and *mbpb*, both include signals that regulate mRNA transport and translation. The mRNA containing the *mbpb* 3’UTR, however, appears to be transported less efficiently towards the myelin sheaths than Dendra2-*mbpa* 3’UTR mRNA (Figure 6J) and endogenous *mbpb* mRNA (Figure 6I), resulting in a more dotted appearance of the WISH signal (Figure 6J). Further analysis of mRNA transport in individual cells is required to confirm such differences.

**Figure 7:**
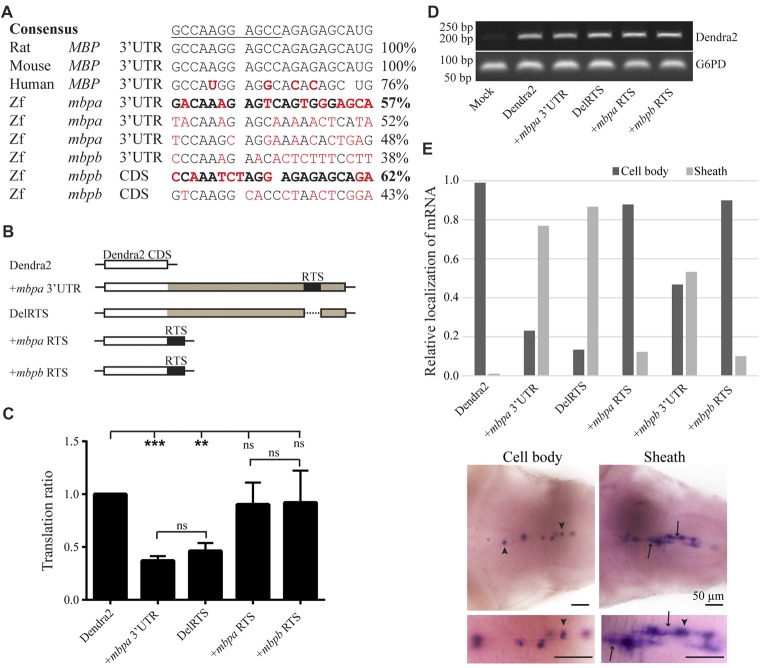
The proposed RNA trafficking sequence (RTS) sequences from *mbpa* and *mbpb* do not affect protein translation or mRNA transport. **(A)** The RTS consensus sequence (the hnRNP A2-binding site, A2RE11, is underlined) and the specific sequences of the rat, mouse and human RTS are shown together with proposed RTS sequences from zebrafish *mbpa* and *mbpb* mRNA. Nucleotides differing from the consensus are marked in red. The sequences from *mbpa* and *mbpb* with the highest similarity to the consensus are marked in bold. **(B)** The constructs tested in the translation assay are illustrated. The *mbpa* and *mbpb* RTS corresponds to the sequences marked in bold in **(A)**. **(C)** The constructs shown in **(B)** were co-transfected with a DsRed-expressing vector into Oli-Neu cells and the translation ratio calculated as described in the “Materials and Methods” section. Data represent the means of four individual experiments ± SD. ns = not significant, **p < 0.01, ***p < 0.001. **(D)** RNA was extracted from a fixed number of transfected cells and RT-PCR was performed with primers specific for *Dendra2* or *G6PD* (control). **(E)** The localization of mRNA in larvae with mosaic expression of the indicated constructs was defined as “Cell body” or “Sheath”. Examples of both groups are shown below the chart. Cell bodies are marked with arrowheads, while sheaths are marked with arrows. Bottom micrographs are enlarged views of top images. The relative fraction of cells with each type of mRNA distribution is plotted. For +*mbpb* RTS, 10 cells were analyzed. For all other constructs, 77–173 cells were analyzed.

As the translation inhibitory function of the *Mbp* 3’UTR was found to be conserved in zebrafish, we asked whether the RNA transport signal (RTS), known from cell-based studies to be important for *Mbp* mRNA transport in mammals (Ainger et al., 1997), was also conserved. A sequence within the 3’UTR of *mbpa* mRNA with significant similarity to the RTS consensus sequence has been identified (Brösamle and Halpern, 2002), however, the function of this proposed RTS has not been investigated. By searching for RTS-like motifs in the *mbpa* and *mbpb* mRNA sequences, additional regions with moderate similarity to the RTS were identified (Figure 7A). For *mbpa*, the previously proposed RTS (Brösamle and Halpern, 2002) had the highest sequence identity with the consensus sequence. Within this motif, 8 out of the 11 nucleotides of the minimal hnRNP A2-binding sequence (A2RE11) were completely conserved (Figure 7A). For *mbpb*, a motif conforming to the RTS consensus sequence in the 3’UTR could not be identified. Therefore, we expanded our search to also include the 5’UTR and coding sequence of *mbpb* and discovered a 21-nt stretch within the coding region with a sequence identity of 62% to the consensus RTS (Figure 7A). As the 3’UTR of *mbpb* alone was found to induce mRNA transport (Figure 6G), other transport elements are likely to be present in this region. These elements remain to be identified.

The potential function of the proposed RTS-like motifs was first assessed in a cell-based translation assay. The translational efficiency of reporter constructs where the RTS was either deleted from the *mbpa*-3’UTR, or the *mbpa* or *mbpb* RTS sequence was inserted directly after a fluorescent reporter protein (Figure 7B), was measured by flow cytometry. Results showed that deletion of the RTS-like motif from the *mbpa*-3’UTR did not relieve the translational inhibitory effect exerted by this region (Figure 7C). Moreover, addition of the RTS-like sequences alone did not affect translation (Figure 7C). RT-PCR analyses demonstrated that all constructs were transcribed at similar levels (Figure 7D), thereby showing that the proposed RTS-like motifs, similar to what we observed previously for the rat RTS sequence, (Torvund-Jensen et al., 2014), are not involved in translational regulation.

Next, we asked whether the RTS-like motifs with the highest similarity to the mammalian RTS were sufficient to drive mRNA localization. To address this question, reporter constructs were placed under the control of the *mbpa* promoter in Tol2 vectors. The vectors were co-injected into fertilized zebrafish embryos with transposase mRNA and the larvae screened for mosaic Dendra2-expression at 78 hpf. WISH was performed on larvae with a specific expression of Dendra2 in the CNS to visualize the localization of the different mRNAs. The localization of mRNA in individual cells was defined as either “cell body” or “sheath” (Figure 7E). *Dendra2* mRNA was found to be restricted to oligodendrocyte cell bodies, while +*mbpa* 3’UTR mRNA was also observed in the myelin sheaths (Figure 7E). In contrast, the *+mbpb* 3’UTR mRNA was only localized to the myelin sheaths in approximately half of the positive cells. This suggests either that there is a difference in the transport efficiency between *mbpa* and *mbpb* mRNAs, or that additional motifs involved in the localization of *mbpb* may be found within its 5’ UTR or its coding sequence. The endogenous *mbpa* and *mbpb* transcripts seem to be transported similarly (Figures 6B,C,E,F,H,I), supporting the existence of additional transport motifs.

Interestingly, when the RTS was deleted from the *mbpa* 3’UTR (DelRTS), the mRNA still localized to the myelin sheaths. Furthermore, when the 21 nucleotides of the predicted *mbpa* or *mbpb* RTS were added to the reporter mRNA, most mRNA was still restricted to the cell body (Figure 7D). Taken together, the data suggests that at least in zebrafish, transport and localization of the mRNA is not dependent on the motifs with highest sequence similarity to the consensus RTS. Combined with the observation that efficient targeting of the *mbpb* mRNA requires elements found outside the 3’UTR, this implicates that several elements function together to ensure targeting to the myelin sheath.

### Neuronal Activity Regulates Mbp Expression in a 3’UTR Dependent Manner

**Figure 8:**
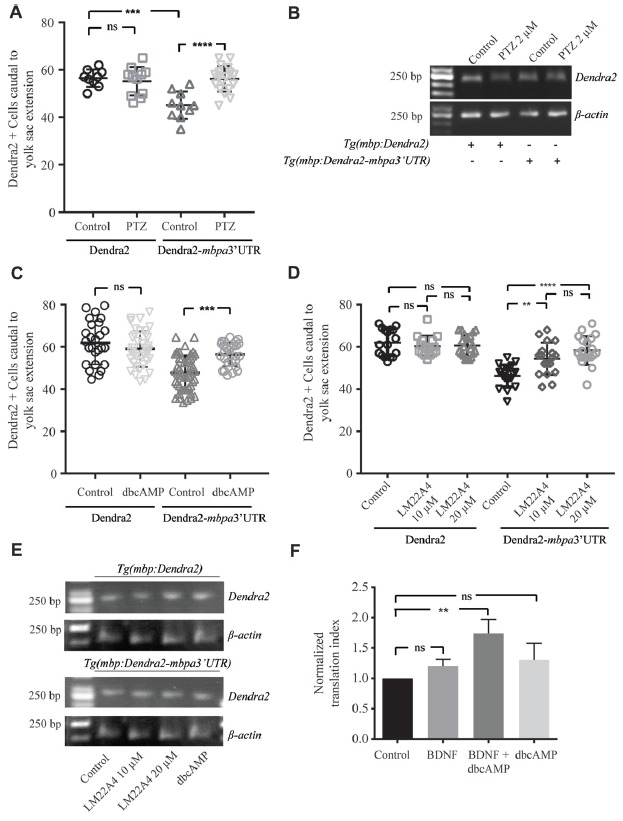
Neuronal activity regulates translation of MBP mRNA *in vivo*. **(A)** *Tg(mbpa:Dendra2)* and *Tg(mbpa:Dendra2-mbpa3’UTR)* were treated with pentylenetetrazole (PTZ; 2 mM) at 72 hpf. At 96 hpf the number of Dendra2 cells caudal to the yolk sac extension was quantified. Mean ± SD is displayed. ns = not significant, ***p < 0.001, ****p < 0.0001. **(B)** *Tg(mbpa:Dendra2)* and *Tg(mbpa:Dendra2-mbpa3’UTR)* were treated with PTZ (2 mM) at 72 hpf. At 96 hpf mRNA was isolated from the larvae and the expression of Dendra2 and actin mRNA was analyzed by RT-PCR. **(C)** *Tg(mbpa:Dendra2)* and *Tg(mbpa:Dendra2-mbpa3’UTR)* were treated with dbcAMP (10 mM) at 72 hpf. At 96 hpf the number of Dendra2 cells caudal to the yolk sac extension was quantified. Mean ± SD is displayed. ns = not significant, ***p < 0.001 **(D)** *Tg(mbpa:Dendra2)* and *Tg(mbpa:Dendra2-mbpa3’UTR)* were treated with 10 or 20 μM of the TrkB agonist LM22A4 at 72 hpf. At 96 hpf the number of Dendra2 cells caudal to the yolk sac extension was quantified. Mean ± SD is displayed. ns = not significant, **p < 0.01, ****p < 0.0001. **(E)** *Tg(mbpa:Dendra2)* and *Tg(mbpa:Dendra2-mbpa3’UTR)* were treated with dbcAMP (10 mM) or LM22A4 (20 μM) at 72 hpf. At 96 hpf mRNA was isolated from the larvae and the expression of Dendra2 and actin mRNA was analyzed by RT-PCR. **(F)** Oli-neu cells were co-transfected with DsRed and Dendra2-pcDNA3.1 or Dendra2-mbpa3’UTR-pcDNA3.1. Twenty-four hours after transfection the cells were treated with dbcAMP (1mM), BDNF (20 ng/mL) or a combination hereof. Forty-eight hours after transfection, expression was analyzed by flow cytometry and the relative changes in expression were calculated. Mean ± SD of at least three independent experiments is plotted. ns = not significant, **p < 0.01.

Based on *in vitro* experiments, neuronal activity has been suggested to be involved in the regulation of MBP translation (Wake et al., 2011). To test this *in vivo*, we treated zebrafish with pentylenetetrazole (PTZ), which has previously been show to enhance neuronal activity in this organism (Mensch et al., 2015). Following short term treatment with PTZ, an increased number of Dendra2-positive cells was observed in *Tg(mbpa:Dendra2-mbpa3’UTR)* larvae, but not in*Tg(mbpa:Dendra2)* larvae (Figure 8A). The levels of *Dendra2* mRNA were not affected by the treatment (Figure 8B). To further identify signals mediating this effect, similar experiments were conducted with dbcAMP and a TrkB agonist (LM22 A4). These compounds were selected as oligodendrocytes have previously been shown to respond to neuronal activity in a cAMP-dependent manner (Malone et al., 2013). Furthermore, the TrkB ligand, BDNF, is known to be released from electrically stimulated neurons (Balkowiec and Katz, 2002). In both cases, an increase in the number of Dendra2-positive cells was observed in the *Tg(mbpa:Dendra2:mbpa3’UTR)* larvae, whereas no effect was observed in *Tg(mbpa:Dendra2)* larvae (Figures 8C,D). Similar levels of *Dendra2* mRNA were found in treated and untreated larvae in both strains (Figure 8E). Combined, this implicates that both cAMP- and TrkB-signaling could be involved in activity-dependent regulation of *mbp* mRNA translation. To confirm that these signals acted directly on the oligodendrocytes, cell-based translation assays were performed as described previously, but in the presence of dbcAMP and/or BDNF. Interestingly, no significant effect was observed following these treatments individually (Figure 8F). However, when the cells were stimulated with both dbcAMP and BDNF, an increase in the translation index was observed (Figure 8F). This implicates that several signals are required to initiate *mbp* mRNA translation and that neurons or other surrounding cells may provide these signals *in vivo*.

## Discussion

During recent years, the zebrafish has emerged as a useful *in vivo* model for studying myelination (Preston and Macklin, 2015; Czopka, 2016). Initial studies have demonstrated that Mbp is expressed in zebrafish and has a distribution within myelin sheaths similar to what is observed in mammals (Brösamle and Halpern, 2002; Nawaz et al., 2013). However, details concerning expression of specific isoforms and post-transcriptional regulation in zebrafish are lacking. In this study, we characterized the expression patterns of the different *mbpa* and *mbpb* isoforms and we used a combination of cell-based assays and transgenic zebrafish to show that the 3’UTR of both *mbpa* and *mbpb* contains translational regulatory elements. The zebrafish system was further used to analyze the role of the 3’UTRs and potential regulatory elements in mRNA transport. Finally, we used the zebrafish model to show that neuronal activity can reverse the inhibitory effect of the 3’UTR on translation and we identified BDNF as a potential mediator of this effect.

We initially demonstrated that the expression of *mbpa* isoforms differ between the PNS and CNS. Specifically, transcripts including exon-5 were detected exclusively in the PNS. In mammals, *MBP* is also found as several isoforms and the expression of splice variants is developmentally regulated (Barbarese et al., 1978; Carson et al., 1983). Different functions have been assigned to the specific isoforms in mammals. The exon-II-positive isoforms shuttle between the nucleus and cytoplasm in OPCs (Pedraza et al., 1997) and have been suggested to be involved in regulation of cell proliferation (Ozgen et al., 2014), while exon-II-negative isoforms exert the classical functions of MBP in the myelin sheath (Harauz et al., 2009). In zebrafish, both the longest *mbpa* isoform, encoded by *mbpa*-211 (exons 1-2a-2b-3-4-5-6), and the shortest isoform, *mbpa*-209 (exons 1-2a-2b-3-6), have experimentally been confirmed to be associated with the cell membrane in Oli-Neu cells (Nawaz et al., 2013), but so far there is no experimental evidence of a nuclear localized form. Further analysis will be required to delineate the specific function of the different MBP isoforms both in mammals and in zebrafish.

We also demonstrated that the expression of both *mbpa* and *mbpb* increased dramatically in zebrafish from 2 days to 3 days post fertilization. The developmentally regulated expression of *mbpa* is in agreement with previous studies (Brösamle and Halpern, 2002; Buckley et al., 2010; Nawaz et al., 2013). A high and robust expression of *mbpb* has earlier been suggested to occur as early as 11 hpf (Nawaz et al., 2009), however, the primers used for RT-PCR by Nawaz et al. recognized *golli* isoforms (exon 3b), which were also detected at high levels early in development in our study. Therefore, our data demonstrates that the expression of the “classic” *mbpb* isoforms is developmentally regulated in a similar manner to *mbpa*. The timing for upregulation of expression of both *mbp* genes correlates with the onset of myelination.

Similar to what we know from mammals, and in agreement with previously published results (Nawaz et al., 2013; Brösamle and Halpern, 2002), we found that endogenous *mbpa* and *mbpb* mRNAs were localized to myelin sheaths. As the addition of the *mbpa* 3’UTR to a reporter gene resulted in a cellular distribution of mRNA in zebrafish that was indiscernible from that of endogenous *mbpa* and *mbpb*, we conclude that the *mbpa* 3’UTR contains all necessary signals to regulate *mbpa* mRNA localization. The *mbpb* 3’UTR also clearly promoted mRNA transport, although not as efficiently as the *mbpa* 3’UTR, with a higher fraction of mRNA detected in the cell bodies. This indicates that the *mbpb* mRNA contains additional motifs involved in the regulation of mRNA localization in either the 5’UTR or coding region.

Brösamle et al. identified a motif within the *mbpa* 3’UTR with sequence similarity to the RNA transport signal (RTS), known to mediate mRNA transport in mammals through interactions with hnRNP A2 (Hoek et al., 1998; Brösamle and Halpern, 2002). We investigated the effects of deleting this proposed RTS from the *mbpa* 3’UTR on the translation and localization of mRNA. Interestingly, we were not able to detect any difference, neither in the translational repression nor mRNA transport exerted by the 3’UTR as a result of this deletion. However, a minor effect on mRNA localization was observed when the proposed *mbpa* RTS was added directly after the coding region of a reporter gene. Previous experiments performed with rat *Mbp* mRNA demonstrated that the RTS alone was sufficient for mRNA transport into the processes of the oligodendrocyte, but additional motifs were required for the localization to myelin sheaths (Ainger et al., 1997). In studies aiming to investigate the role of the RTS in mammals, the RTS has not been deleted specifically from the 3’UTR. Instead, the effect of a short region containing the RTS (Ainger et al., 1997) or the isolated RTS were investigated (Munro et al., 1999; Shan et al., 2000; Kosturko et al., 2006). Therefore, it cannot be excluded that the 3’UTR of mammalian *MBP* mRNAs with a specific deletion of RTS could also support mRNA transport. As the RTS-like motifs are not crucial for *mbp* mRNA localization in zebrafish we suggest that there are other regulatory motifs involved in targeting the mRNA to the myelin sheath, in resemblance to mammalians. These may include alternative hnRNP A2-binding motifs, such as the conditional GA-type RNA targeting motif recently reported to interact with hnRNP A2 in a calcium-dependent manner (Muslimov et al., 2014). The identification of such motifs and corresponding trans-acting factors will be important to obtain a more complete understanding of how *MBP* mRNA transport is regulated as the current model is based exclusively on an essential role of hnRNP A2 and its interaction with the RTS. Other recent experiments also suggest that transport of the MBP mRNA is complex and may involve multiple steps of intracellular transport, as both a kinesin in the form of Kif1b (Lyons et al., 2009) and a dynein/dynactin complex (Herbert et al., 2017) are required for targeting of the mRNA to the myelin sheath.

We further showed that the 3’UTRs from both zebrafish *mbpa* and *mbpb* mRNAs were able to inhibit protein translation both in zebrafish and in cultured cells of murine origin. This suggests that both 3’UTRs include regions recognizable by transacting factors from different species, despite a difference in size of approximately 800 nucleotides between the two and a sequence identity of only approximately 30% (analyzed with the EMBOSS Needle tool^1^). The conserved function but low overall similarity likely reflects that many of the cis-regulatory elements are relatively short sequence motifs and in many cases the interaction with trans-regulatory elements also depends on the secondary structure of the mRNA.

In zebrafish, protein translation was quantified by counting the number of Dendra2-positive cells in an area where new cells are gradually appearing as a result of normal differentiation, i.e., not by quantifying the amount of expressed fluorescent protein. By this approach, the effect of the 3’UTR on the *onset* of protein translation in individual cells is assessed. When comparing hetero- and homozygotic transgenic larvae, no difference in the number of Dendra2-positive cells was observed within a specific transgenic strain. This further suggests that the 3’UTRs, not the amount of mRNA, control the timing of Mbp protein expression in individual oligodendrocytes. This supports the model where reversal of the inhibitory effect of the 3’UTR is a key regulatory step to control initiation of translation of the MBP mRNA. However, we cannot exclude that additional regulatory steps are in place to control the amount of MBP produced locally.

In a final set of experiments, we used the zebrafish model system to identify signals involved in regulating the onset of *mbp* mRNA translation. We found that short-term pharmacological stimulation of neuronal activity with PTZ, reversed the inhibitory effect of the 3’UTR on reporter protein expression without altering the mRNA levels. Previous studies by others have shown that PTZ stimulates myelination in the zebrafish (Mensch et al., 2015), but the specific processes activated in the oligodendrocyte to facilitate this effect are not known. Our results suggest that one mechanism by which neuronal activity can stimulate myelination is by regulating the onset of local Mbp translation. We further identified signaling pathways that may be mediating this effect. Stimulation with a TrkB agonist mimicking signals released from neurons (BDNF), and dbcAMP also reversed the inhibitory effect of the *mbpa* 3’UTR on protein expression. To determine whether these signals were acting directly on the oligodendrocyte we performed similar experiments in cell cultures. Interestingly, a combination of BDNF and a cyclic AMP analog was required to enhance the expression of reporter constructs in the oligodendrocyte precursor cell line. This suggests that the signals are working directly on the oligodendrocytes. This also highlights the importance of working with an *in vivo* system, as screening for regulatory factors in the cell-based system would not have revealed an effect of the individual factors. Altogether, this implicates that several signals from neurons or other cell types are required to initiate MBP translation. Synaptic activity has been reported to stimulate local translation in neuronal dendrites; however, the specific mechanistic details enabling local regulation remain unclear. BDNF signaling has been shown to induce phosphorylation of Akt, leading to activation of the mTOR pathway (Takei et al., 2004), but these signals most likely result in a general increase in translation, not necessarily restricted to the dendrites. Other studies have shown that BDNF signaling is involved in recruiting specific hnRNP molecules to dendritic spines. Of particular interest for MBP translation, these include hnRNP K (Leal et al., 2017), hnRNP A2 (Leal et al., 2014) and CBF-A (Raju et al., 2011), all transacting factors known to be associated with the *MBP* mRNA (Hoek et al., 1998; Raju et al., 2008; Laursen et al., 2011). Furthermore, neuronal activity has been suggested to induce a partial rearrangement of messenger ribonucleoproteins (mRNPs), which suggests a release of mRNA for translation (Park et al., 2014). Based on this, one possible function of BDNF released from active neurons could be to recruit hnRNP K to the growing myelin sheaths, thereby promoting a local rearrangement of the MBP mRNP in which translational inhibitory hnRNP E1 is exchanged with the stimulatory hnRNP K (Torvund-Jensen et al., 2014). Such hypothesis needs to be tested in future studies, aiming to determine how different axonal signals are integrated to coordinate transport and local translation of *MBP* mRNA in the oligodendrocyte. Our experiments suggest the zebrafish as a model system to study these mechanisms *in vivo* and implicate BDNF- and cAMP-dependent signaling pathways as novel regulators.

## Author Contributions

JT-J, JS, KK-S and LL designed the experiments. JT-J, JS, LA and LL performed the experiments. JT-J, JS and LL analyzed the data. JT-J and LL wrote the manuscript with input from KK-S and JS.

## Conflict of Interest Statement

The authors declare that the research was conducted in the absence of any commercial or financial relationships that could be construed as a potential conflict of interest.

## Footnotes

^1^http://www.ebi.ac.uk/Tools/psa/emboss_needle/
