# Supplementary figures and images for: The 3’UTRs of Myelin Basic Protein mRNAs Regulate Transport, Local Translation and Sensitivity to Neuronal Activity in Zebrafish
Source: Front Mol Neurosci. 2018 Jun 12;11:185. doi: 10.3389/fnmol.2018.00185 (PMC6006989; doi:10.3389/fnmol.2018.00185)

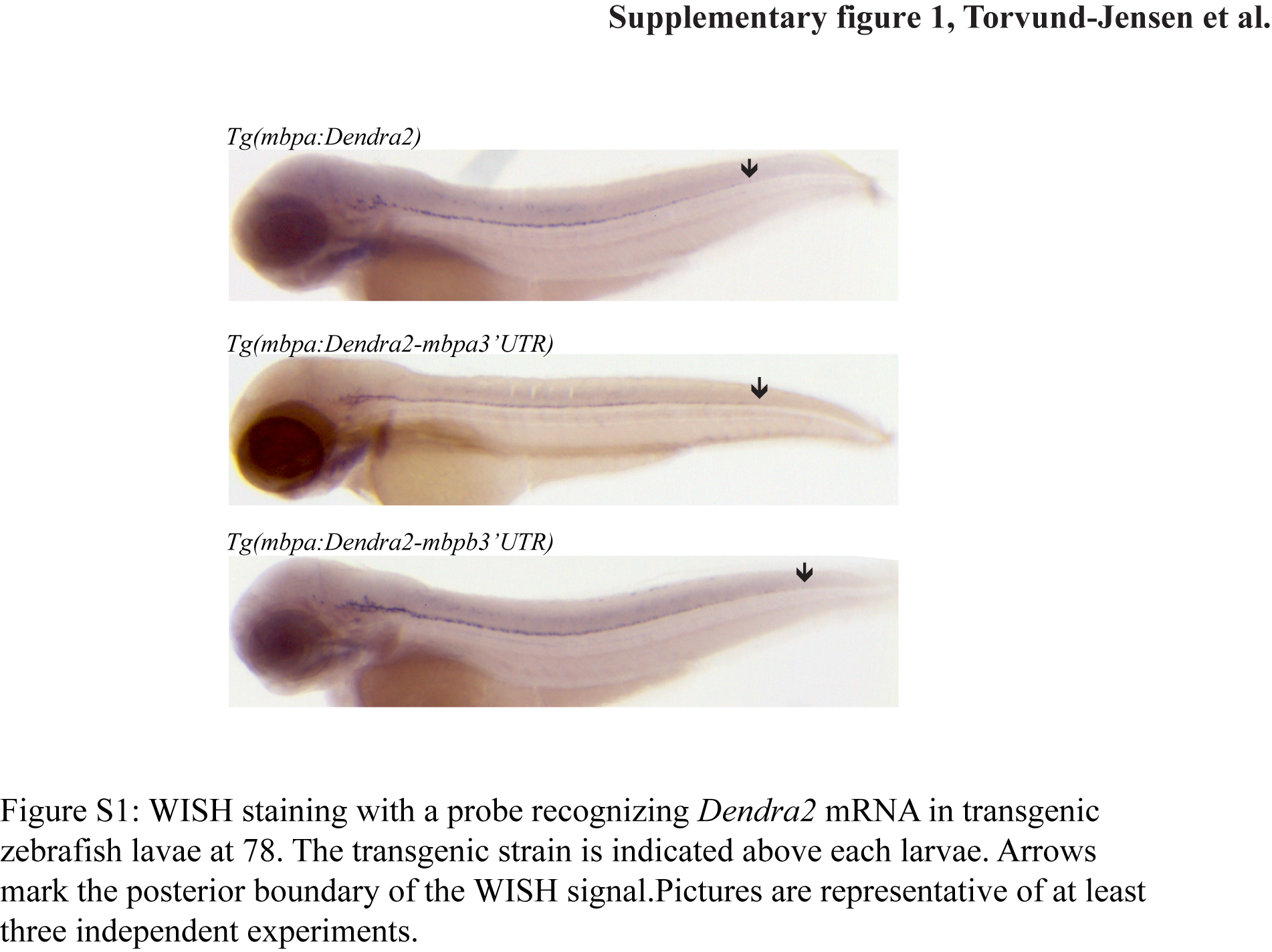

Supplement: Supplementary file 1 [file Image_1.TIF]

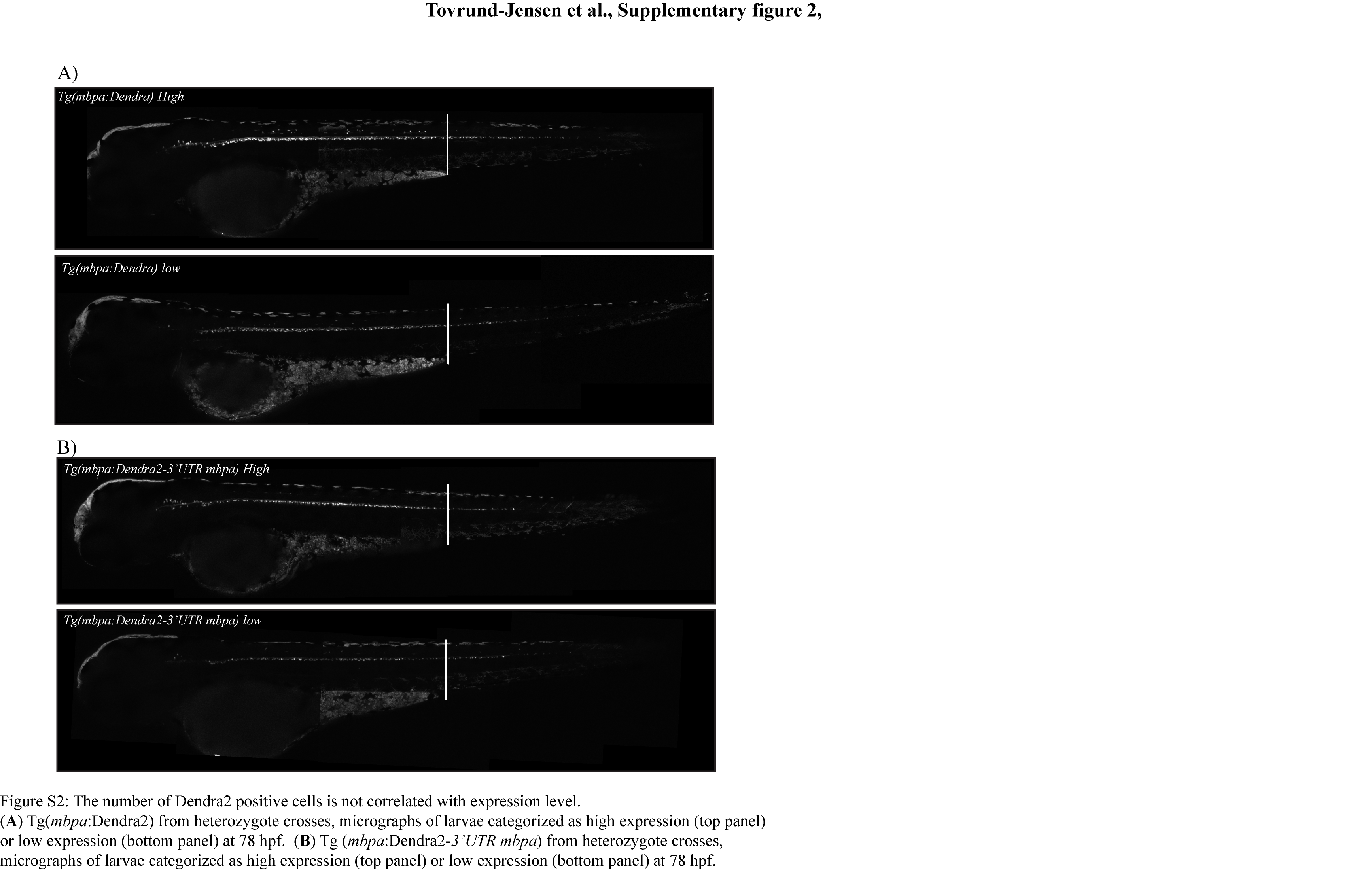

Supplement: Supplementary file 2 [file Image_2.tif]

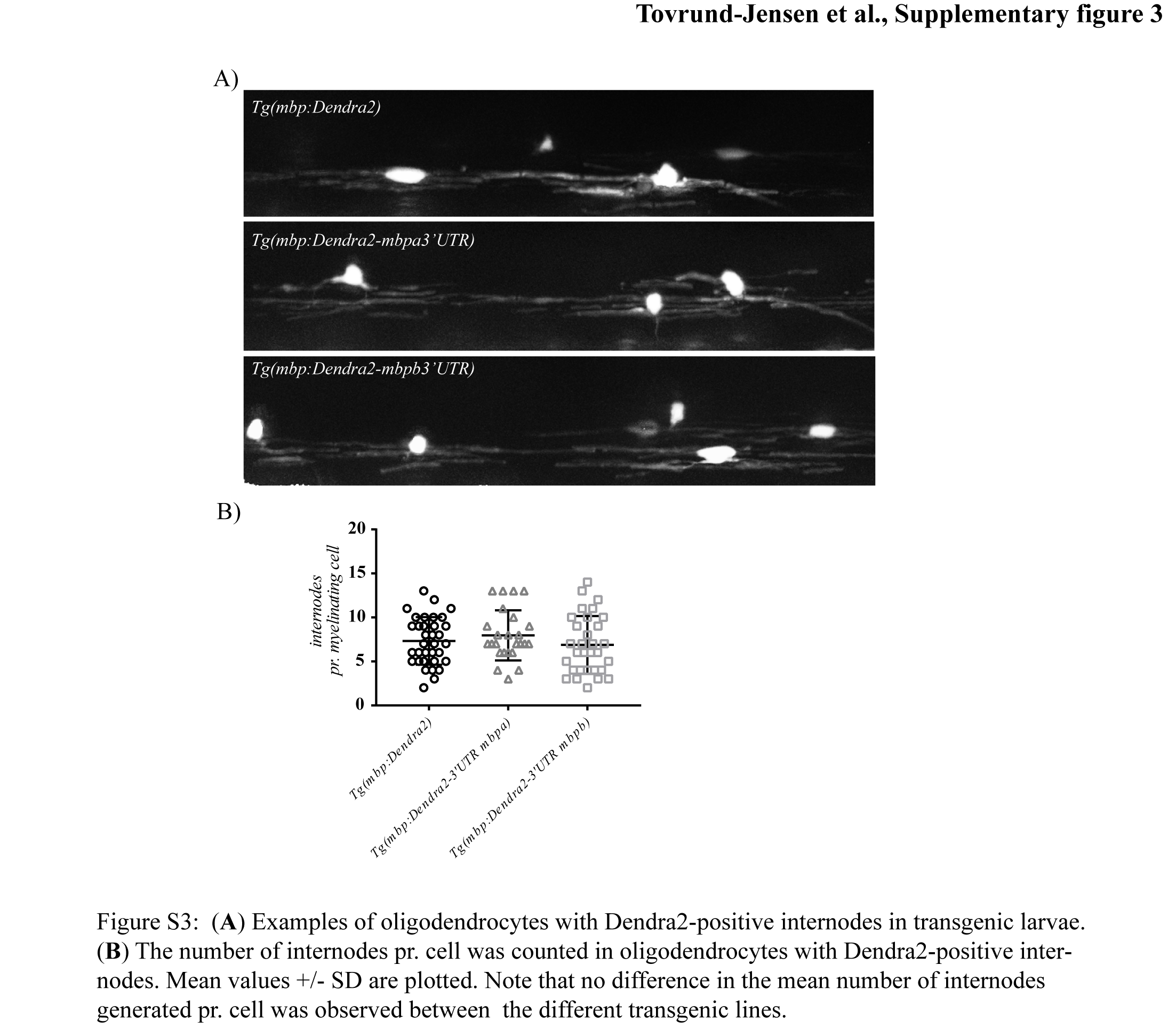

Supplement: Supplementary file 3 [file Image_3.tif]
